# Hybrid Sequencing of Full-Length cDNA Transcripts of Stems and Leaves in *Dendrobium officinale*

**DOI:** 10.3390/genes8100257

**Published:** 2017-10-05

**Authors:** Liu He, Shuhua Fu, Zhichao Xu, Jun Yan, Jiang Xu, Hong Zhou, Jianguo Zhou, Xinlian Chen, Ying Li, Kin Fai Au, Hui Yao

**Affiliations:** 1 Key Laboratory of Bioactive Substances and Resources Utilization of Chinese Herbal Medicine, Ministry of Education, Institute of Medicinal Plant Development, Chinese Academy of Medical Sciences & Peking Union Medical College, Beijing 100193, China; lhe@implad.ac.cn (L.H.); xuzhichao830@126.com (Z.X.); zhouhong1013@126.com (H.Z.); jgzhou1316@163.com (J.Z.); chenxinlian1053@163.com (X.C.); liying@implad.ac.cn (Y.L.); 2 Department of Internal Medicine, University of Iowa, Iowa City, IA 52242, USA; shuhua-fu@uiowa.edu; 3 Department of Plant Genetic Breeding, College of Agriculture and Biotechnology, China Agricultural University, Beijing 100193, China; yjcbscau@126.com; 4 Institute of Chinese Materia Medica, Academy of Chinese Medical Sciences, Beijing 100700, China; jxu@icmm.ac.cn; 5 Department of Biostatistics, University of Iowa, Iowa City, IA 52242, USA

**Keywords:** *Dendrobium officinale*, single-molecule real-time sequence, SMRT, second-generation sequence, SGS, polysaccharide, sugar transporter, alternative splicing

## Abstract

*Dendrobium officinale* is an extremely valuable orchid used in traditional Chinese medicine, so sought after that it has a higher market value than gold. Although the expression profiles of some genes involved in the polysaccharide synthesis have previously been investigated, little research has been carried out on their alternatively spliced isoforms in *D. officinale*. In addition, information regarding the translocation of sugars from leaves to stems in *D. officinale* also remains limited. We analyzed the polysaccharide content of *D. officinale* leaves and stems, and completed in-depth transcriptome sequencing of these two diverse tissue types using second-generation sequencing (SGS) and single-molecule real-time (SMRT) sequencing technology. The results of this study yielded a digital inventory of gene and mRNA isoform expressions. A comparative analysis of both transcriptomes uncovered a total of 1414 differentially expressed genes, including 844 that were up-regulated and 570 that were down-regulated in stems. Of these genes, one sugars will eventually be exported transporter (SWEET) and one sucrose transporter (SUT) are expressed to a greater extent in *D. officinale* stems than in leaves. Two glycosyltransferase (GT) and four cellulose synthase (Ces) genes undergo a distinct degree of alternative splicing. In the stems, the content of polysaccharides is twice as much as that in the leaves. The differentially expressed GT and transcription factor (TF) genes will be the focus of further study. The genes *DoSWEET4* and *DoSUT1* are significantly expressed in the stem, and are likely to be involved in sugar loading in the phloem.

## 1. Introduction

The perennial epiphytic herb *Dendrobium officinale* Kimura et Migo is a rare lithophytic orchid that lives in dark and damp rocky environments in Chinese subtropical mountainous regions. Dendrobii Officinalis Caulis, the dried stem of *D. officinale*, has been used as a tonic in traditional Chinese medicine (TCM) for thousands of years to diminish inflammation, counteract senescence, and improve immunity [1]. Water-soluble polysaccharides are the dominant active ingredients in *D. officinale* [2,3,4,5], comprising glucose, mannose, galactose, xylose, and arabinose in different proportions [6,7]. As carbohydrates are produced in green plant leaves as a result of photosynthetic activity, assimilated carbon is moved immediately out of the leaves and is mainly converted to polysaccharides in the stem and roots [8].

The *D. officinale* genes associated with polysaccharide synthesis, including glucose transporters (GT) and glycoside hydrolases, have been evaluated in previous studies [9,10,11]. In addition to polysaccharide synthesis, sugar translocation from the leaves to the stems is particularly noteworthy, because the sugar content of plants generally depends on metabolism and transport [12].

Sucrose is actively transported from the leaves to other tissues to sustain growth and metabolism. SWEET (sugars will eventually be exported transporters) refers to a new class of transporters that are essential for the movement of sugars between cells in both plants and animals [13]. Investigations have shown that SWEETs are widespread in plants; 17 have been identified in *Arabidopsis thaliana*, 21 in *Oryza sativa*, 23 in *Sorghum bicolor*, and 29 in *Solanum lycopersicum* [13,14,15].

In *A. thaliana*, the SWEETs of sucrose, glucose, galactose, fructose, and 2-deoxyglucose have been shown to act as bidirectional transporters, mediating both sugar uptake and efflux across the plasma membrane in leaves, roots, flowers, pollens, and seeds [13,16,17]. In rice, for example, the transporters OsSWEET11 and OsSWEET14 participated in the efflux of photosynthesized sucrose from the leaves [13,18], with the former required for pollen development. In addition to SWEETs, sucrose transporters (SUTs) are also responsible for sucrose importation from the apoplast into phloem cells; ZmSUT1, for example, is known to be responsible for sucrose loading and unloading in the phloem [19,20]. Despite this background, however, information regarding sugar accumulation in the stem remains limited as the model plants investigated to date accumulate just small amounts of these carbohydrates in this region. Nevertheless, a larger polysaccharide volume has been identified in stems of *D. officinale* [1]; but the molecular mechanisms that underlie this accumulation necessitate further investigation.

Because of interest in the medicinal properties of *D. officinale*, extensive DNA and RNA sequencing studies have been carried out recently on this extremely valuable plant [9,10,21,22]. However, molecular information on the differing gene expression in relation to sugar translocation between the leaves and stems of *D. officinale* remains limited. The advent of PacBio sequencing has meant that full length gene transcripts are now available while the use of second generation sequencing (SGS) has led to improvements in sequence quality. Hybrid sequencing strategies are now generally both more affordable and of higher quality than the use of single sequencing alone; indeed, the combined use of single-molecule real-time (SMRT) long reads and SGS short reads has enabled the detection of sensitive isoforms and predictions based on genome sequences [23,24]. The production of gene isoforms has also led to an increase in the structural and functional variety of gene products.

In this study, we utilized the combination of SGS and SMRT sequencing technology to generate two organ-specific full-length transcriptomes for *D. officinale*. We applied this strategy to dissected leaf and stem samples, thereby enabling more correlation of specific expression information from resultant transcriptional data to stems, where polysaccharides are accumulated. The results of this study are significant for future research on the molecular mechanisms of sugar translocation and polysaccharide accumulation in *D. officinale.*

## 2. Material and Methods

### 2.1. Plant Materials and RNA Sample Preparation

All the *D. officinale* seedlings (three-year-old) were harvested from a plastic greenhouse (Lin’an City, Zhejiang Province, China). A total of forty independent seedlings were collected. The leaves and stem of each seedling were separated and wrapped individually in pieces of aluminum paper, frozen in liquid nitrogen, and stored at −80 °C prior to RNA isolation and polysaccharide extraction. Each leaf (stem) RNA sample came from five seedlings. *Dendrobium fimbriatum* Hook. (three-year-old) leaves and stems were cut from a local greenhouse (Nanning City, Guangxi Province, China). The collection method used is the same as above.

### 2.2. Polysaccharide Determination

We followed the method outlined in the Pharmacopeia of the People’s Republic of China (2015) to determine the polysaccharide content of *Dendrobium nobile*, performing three parallel experiments under the same conditions. The anhydrous d-glucose (batch number 110833–201205) used for all experiments was purchased from the Chinese Food and Drug Inspection Institute, and an initial reference solution was prepared at a concentration of 93 µg/mL.

### 2.3. RNA Preparation and cDNA Synthesis

We isolated total RNA using a RNeasy plus mini kit (#74134, Qiagen, Duesseldorf, Germany). RNA quality was then assessed using EtBr-stained 0.8% agarose gels, while concentration and integrity were evaluated using an Agilent 2100 Bioanalyzer (Agilent, Palo Alto, CA, USA). Transcriptome sequencing was carried out using Illumina Hiseq 1500 (Illumina, San Diego, CA, USA) and PacBio RS II systems (Pacific Biosciences, Menlo Park, CA, USA), following the manufacturer’s instructions. SMRT sequence and Illumina HiSeq 1500 data have been submitted to the Sequence Read Archive (SRA) of the NCBI database under study accession number SRP094520 of bioproject PRJNA356162.

### 2.4. Transcriptome Sequencing Data Analysis

Transcriptome assembly was carried out using all clean data in the software Trinity2 [25]. The parameter min kmer cov was set to 2 and all other parameters were set to default. Open reading frame (ORF) prediction of assembled transcripts was performed using TransDecoder (Find Coding Regions within Transcripts). Gene function was annotated using Trinotate2.0 based on Pfam [26], egg-NOG [27], KEGG [28] and GO [29].

### 2.5. Differential Expression Analysis

Firstly, the read counts were corrected by edgeR [30] program package through one scaling normalized factor. After that, the differential expression analysis of the two samples was performed using the DEGSeq R package (1.12.0) [31]. The Benjamini–Hochberg method was used to adjust the *p*-value. Corrected *p*-value of 0.005 and log2 (Fold change) of 1 were set as the threshold for determining whether expression was significantly differential.

### 2.6. Isoform Detection and Prediction

The quality of short-read libraries was evaluated using the software FastQC 0.11.4 [32], with 13 nucleotides from the 3′ end on each read trimmed. Adapters among short-read libraries were then removed using the software Cutadapt [33], and errors in PacBio long reads were corrected with short-read alignment using the software LSC 1.beta [34]. Following error correction, long and short reads were aligned to the genome using the software HISAT [35], before being input into the software IDP 0.1.9 [23] for the detection and prediction of isoforms.

### 2.7. Real-Time PCR

Two RNA samples were isolated from *D. officinale* stems and leaf tissues and reverse transcription was performed using PrimeScript^TM^ Reverse Transcriptase (TaKaRa, Tokyo, Japan). At the same time, qPCR primers were designed using PRIMER PREMIER 6 (Premier Biosoft International, Palo Alto, CA, USA), and their specificity was verified using PCR. qPCR analysis was then conducted in triplicate using SYBR^®^ Premix Ex Taq^TM^ (TaKaRa, Tokyo, Japan) and a 7500 Real-time PCR system (ABI), with *DoActin* used as the reference gene [36].

### 2.8. Phylogenetic Analysis

We downloaded amino acid sequences for 17 *AtSWEET* and 21 *OsSWEET* genes from the National Center for Biotechnology Information (NCBI) database, pooled them with sequences for eight *DoSWEET* genes, and performed an alignment using the software MEGA 5.0 [37]. An unrooted phylogenetic tree for full-length amino acid sequences was constructed using neighbor-joining and the bootstrap method with 1000 replicates.

## 3. Results

### 3.1. Comparing the Polysaccharide Content of Stems and Leaves

**Figure 1:**
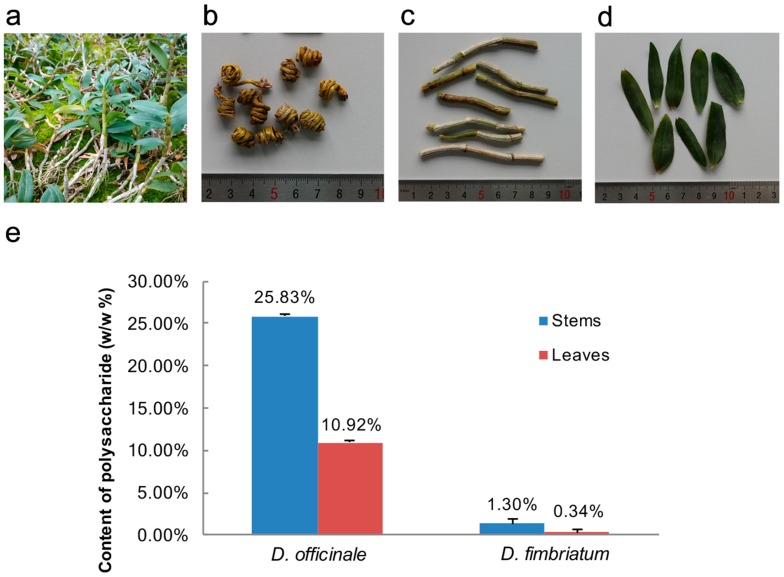
Morphology and chemical profiling of the leaves and stems of *Dendrobium officinale*. (**a**) Cultivar morphology in the greenhouse; (**b**) Morphology of the dry stem used in traditional Chinese medicine (TCM); (**c**) Stems that were sampled for RNA-seq and chemical profiling; (**d**) Leaves that were sampled for RNA-seq and chemical profiling; (**e**) Analyses of the polysaccharide contents of leaves and stems.

As a result of their high polysaccharide content, *D. officinale* plants are used in TCM when they reach heights between 20 cm and 30 cm in the greenhouse (Figure 1a). Indeed, ‘Feng Dou’, the dried stems of *D. officinale* (Figure 1b), have long been used as crude drugs in TCM as they have good efficacy as a tonic. The leaves and stems of seedlings are separated, and polysaccharide contents is detected respectively (Figure 1c,d); the results have shown that the stems of *D. officinale* contain twice as much polysaccharide as the leaves, and that concentrations in *D. officinale* are much higher than in *D. fimbriatum* at the same growth stage (Figure 1e). These differences mean that *D. officinale* is well suited for identifying the key genes involved in sugar translocation between tissues via the phloem. Thus, in order to obtain more information on the molecular mechanisms of sugar accumulation in *D. officinale*, we performed RNA sequencing (RNA-seq) on leaves and stems using SGS and SMRT sequencing platforms.

### 3.2. Hybrid-Seq and Sequence Assembly

**Figure 2:**
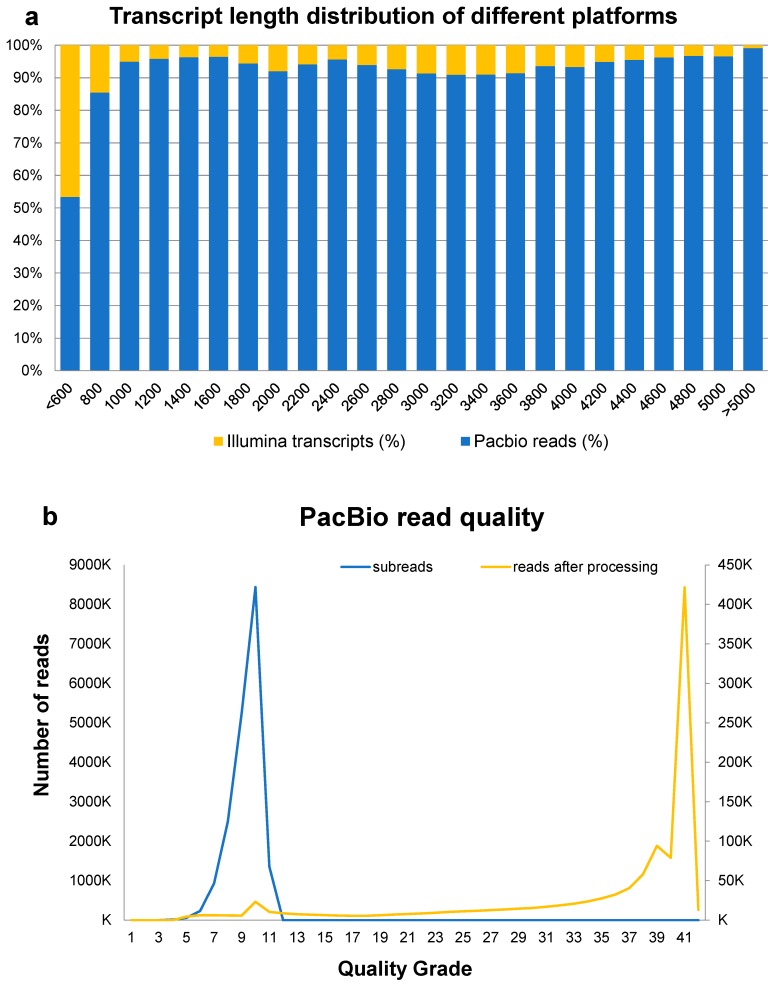
Distribution of *D. officinale* second-generation sequencing (SGS) and single-molecule real-time (SMRT) reads. (**a**) Transcript length distributions from the two different sequencing platforms; (**b**) PacBio quality of subreads and corrected reads.

We subjected two mRNA samples from leaves and stems, respectively, to 2 × 150 paired-end sequencing using the HiSeq 1500 platform, generating 68,977,848 reads. Full-length complementary DNA (cDNA) sequences from the same two mRNA samples were then normalized and subjected to SMRT sequencing using the PacBio RS II platform, generating a total of 19,941,155 pre-filter reads. We then carried out post-filtering using the RS_Subreads.1 protocol in the software PacBio RS, generating 18,772,392 subreads. These were then combined with SGS sequences to improve the number of correct subreads. This process generated a total of 197,029 Illumina screened reads which were then used as input data to generate 321,433 corrected reads. The transcript length distribution generated using these two platforms, as well as the quality of PacBio reads, can be seen in Figure 2. Combining high quality SMRT long reads, 1,083,400 corrected reads were then used as input for further analysis; the use of long SMRT sequencing reads (N50 = 1863 base pairs, bp) generated the full-length transcripts relative to assemblies using just SGS reads (SGS-assembled unigene N50 = 658 bp).

### 3.3. The Reads per Kilobase per Million Mapped Reads Method to Determine Differential Gene Expression between Leaves and Stems

**Figure 3:**
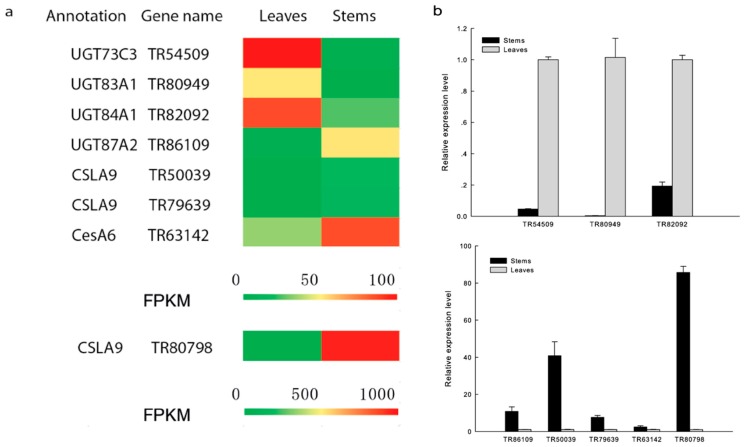
Comparison of polysaccharide biosynthesis candidate gene expression in leaves and stems of *D. officinale*. (**a**) Heat map of candidate gene expression profiles from SGS data; (**b**) RT-qPCR analysis of candidate genes in leaves and stems. Data are shown as means ± standard error (*n* = 3).

The reads per kilobase per million mapped reads (RPKM) method was used to calculate read densities and to compare differences in gene expression between leaves and stems. SGS data show that the use of a RPKM cut-off value greater than 10 reveals that 1414 genes are differentially expressed between leaves and stems (Table S1), and that of these, 844 and 570 are up-regulated in the two tissue types, respectively. Next, we classified the unigenes involved in biosynthetic pathways using KEGG [38,39,40] metabolic categories to enable a more detailed analysis. The results of this classification showed that 161 differentially expressed genes (DEGs) are involved in metabolic pathways; of these, 49, 6, and 12 DEGs belong to the carbohydrate metabolism, secondary metabolism, and glycan biosynthesis and metabolism pathways, respectively. These include three glucomannan 4-beta-mannosyltransferase (CSLA9) genes (TR50039, TR79639, and TR80798), one GT gene (TR86109), and one Ces A catalytic subunit 6 (CesA6) gene (TR63142), all exhibiting higher levels of expression in stems than in leaves (Figure 3a). The high expression level of these transcripts may be linked to the biosynthesis of polysaccharides in *D. officinale*. We also identified three GT genes (TR54509, TR80949, and TR82092) that are up-regulated in leaves, and confirmed all expression levels using real-time PCR (RT-PCR) (Figure 3b). In addition, we classified 37 transcription factor (TF) unigenes as well as 13 TF DEGs that are up-regulated in stems. The expression profiles of these TFs that correlated with genes involved in polysaccharide synthesis or sugar transportation will be a focus of future research.

### 3.4. Candidate Genes for Sugar Translocation in the Stem

**Figure 4:**
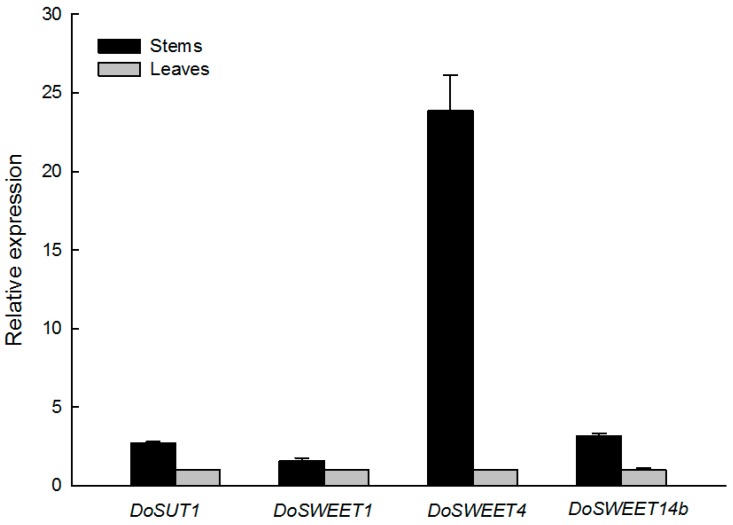
RT-qPCR analysis of *DoSWEETs* in leaves and stems. Data are shown as means ± standard error (*n* = 3).

Sugar transport proteins play crucial roles in both the cell-to-cell and long-distance movement of sugars throughout plants. In particular, both SWEETs and SUTs are intimately involved in sucrose transport; the transcriptome data presented in this study show that three SWEET genes and one SUT gene are expressed to a greater extent in the stems of *D. officinale* than in leaves. We confirmed these levels of differential expression using quantitative RT-PCR. The results of this study show that the genes *DoSWEET1* and *DoSWEET14b* are expressed in stems to about twice the level seen in leaves, while stem expression of *DoSWEET4* is more than 20-fold higher than in leaves (Figure 4).

**Figure 5:**
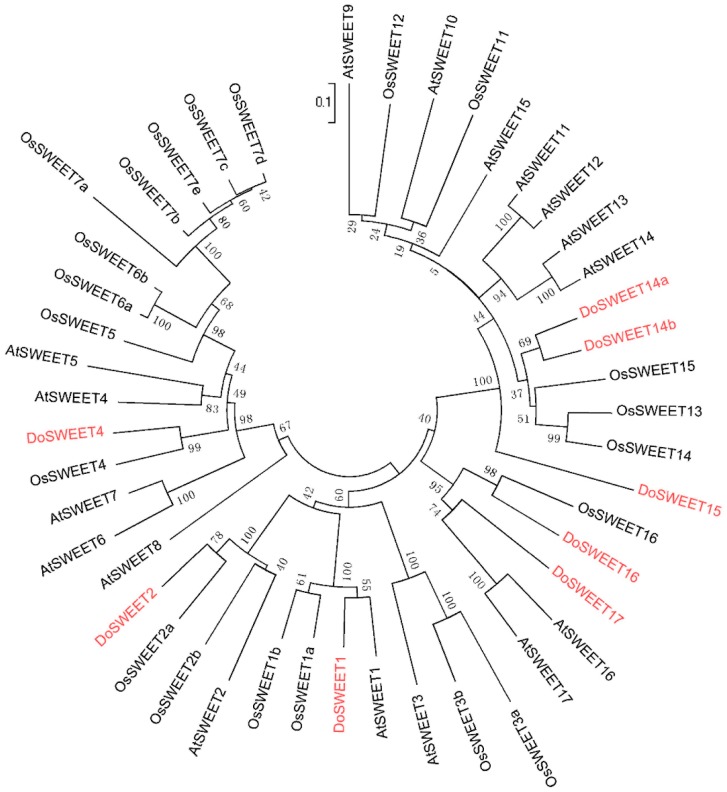
Neighbor-joining phylogenetic tree of sugars will eventually be exported transporters (SWEETs) from *D. officinale*, *Arabidopsis*, and rice. This analysis comprised amino acid sequences for 17 AtSWEETs, 21 OsSWEETs, and 8 DoSWEETs (in red).

Analysis with the software TMHMM 2.0 [41] reveals that the secondary SWEET protein structures of this orchid comprise seven transmembrane helices, while that of SUT contain 12 transmembrane domains (Figure S1). We therefore screened our unigene dataset and, based on annotation results, discovered the presence of five additional *D. officinale* SWEET genes (Table S2). Amino acid sequence alignments were compared phylogenetically with their homologs in *Arabidopsis* and rice using the software MEGA 5.0 [37] (Figure 5), and the new genes identified in this study were named *DoSWEET1*, *DoSWEET2*, *DoSWEET4*, *DoSWEET14a*, *DoSWEET14b*, *DoSWEET15*, *DoSWEET16*, *DoSWEET17*, and *DoSUT1*. Results also reveal similar patterns of expression in *DoSWEET4* compared to *SbSWEET4–3*; both these genes are more significantly expressed in stems than they are in leaves during sucrose accumulation [15]. Indeed, our phylogenetic study shows that *DoSWEET4* and *SbSWEET4–3* cluster together in a single clade with 65.2% identity.

### 3.5. Alternatively Spliced Isoforms

**Figure 6:**
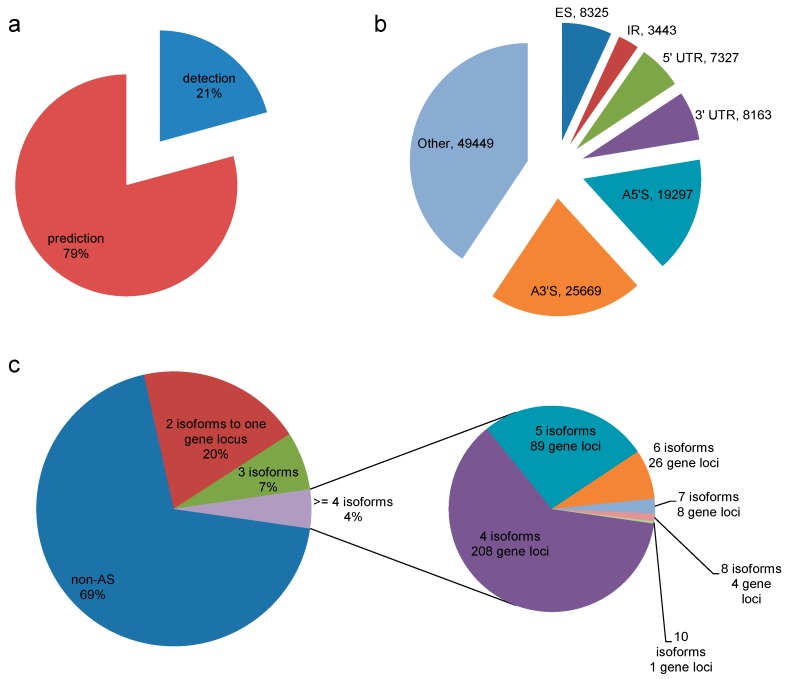
Detection and prediction of *D. officinale* gene isoforms using isoform detection and prediction (IDP). (**a**) Venn diagram to show the detection and prediction of 2680 and 10,230 isoforms, respectively; (**b**) Pie chart to show different alternatively spliced types. ES: exon skipping; IR: intron retention; A3’S: alternative 3′ splice site; A5’S: alternative 5′ splice site; (**c**) The distribution of alternatively spliced isoforms from each gene locus.

Alternative splicing has played an extremely important role in understanding protein diversity [42,43]; full-length transcripts can be used to identify alternative splicing events via the hybrid-seq isoform detection and prediction (IDP) pipeline [23]. IDP analysis combined with the *D. officinale* genome sequence (http://202.203.187.112/genome/dendrobe/) results reveal the presence of 12,910 isoforms in leaves and stem transcriptomes; in total, out of 12,910 multi-exon genes, 2280 (31%) exhibit alternatively spliced isoforms, while two isoforms were found in 19% of cases, three in 7% of cases, and more than four isoforms in 5% of cases (Figure 6). Based on the genome annotation, SMRT sequencing compensates for the defects of SGS assembly strategy in the alternative splicing predicting.

**Figure 7:**
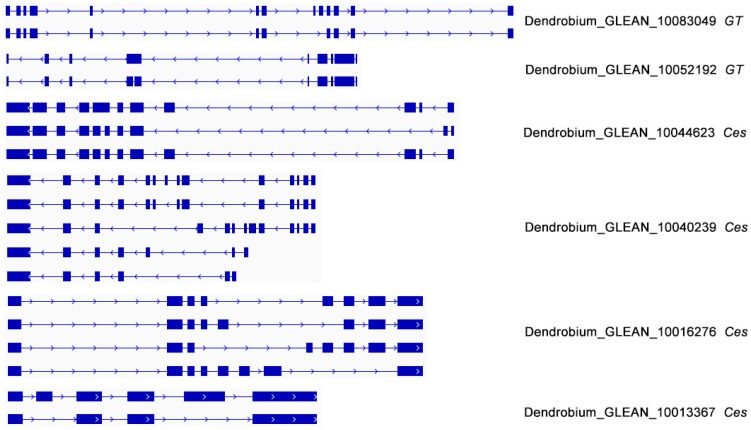
Alternative splicing isoforms of genes involved in polysaccharide biosynthesis encompassed by PacBio long reads. *GT*: glycosyltransferases gene; *Ces*: cellulose synthase gene.

The genes that encode sugar transporters do not seem to undergo alternative splicing to any significant extent, although two GT and four Ces genes, probably involved in polysaccharide metabolism pathways, were identified in the IDP pipeline using a genome-wide approach. Results show that both of these GT genes have two isoforms, while the four Ces genes identified in this study possess five, four, three, and two isoforms, respectively (Figure 7). The alternatively splicing variants possess more than one function. The effect of alternative splicing is not limited to protein isoforms, but extends to gene expression patterns [43].

## 4. Discussion

Natural plant polysaccharides have an extensive history of application in medicine and pharmaceutics. Indeed, polysaccharide bioactivity is increasingly valued in the context of human health. The orchid *D. officinale* has long been used as a highly-prized tonic in TCM, and thus has considerable economic value. Polysaccharide is the main active component in *D. officinale*; the molecular mechanisms underlying bioactive polysaccharide synthesis in this plant have attracted considerable research attention. In earlier work, Zhang and colleagues reported a comparison of transcriptomes from two development stages of *D. officinale*, generated using an Illumina HiSeq™ 2000 platform [10]. Comparative analysis of the transcriptomes of juvenile seedlings and adult plants revealed that numerous candidate synthases are involved in polysaccharide biosynthesis. Thus, to further address the question of polysaccharide synthesis and sugar translocation from leaves to stems, the short-read SGS and long-read SMRT sequencing of the two distinct tissue transcriptomes available for *D. officinale* were combined in this study. Our results show that the expression levels of the genes *DoSWEET4* and *DoSUT1* are markedly increased in stems compared to leaves. Previous work has shown that the gene *ZmSUT1* is critical for the efficient loading of sucrose in the phloem in maize leaves [19]. Thus, *SWEET4* genes that encode hexose transporters in maize and rice might be also responsible for sugar transport along the phloem unloading path [44]. *DoSWEET4* and *DoSUT1* potentially act synergistically in the process of sugar translocation from the leaves to the stems when monosaccharides are combined to form polysaccharides. As discussed, while SWEETs comprise a group of newly identified sugar transporters in *Arabidopsis* and rice [13], this study is the first to report the presence of these transporters in a medicinal plant that is rich in water-soluble polysaccharides.

Sucrose is exported from leaves to stems to sustain plant growth and metabolism; redundant sugars are stored as soluble polysaccharides in the stems. In this respect, *D. officinale* is a typical mycorrhizal plant, dependent on fungi for seed germination and growth during its seedling and early growth stages. The abundant sugars present in the stem of this plant are therefore likely to sustain the growth of mycorrhizal fungi associated with *D. officinale*; these fungi obtain energy from plants in exchange for inorganic substances and other nutrient elements. This form of symbiosis is a key environmental adaptation, and SWEETs are likely to play a significant role in providing mycorrhizae with sugar.

In summary, this study reports the functional genomics of a combination of SGS short-read and SMRT long-read transcriptomes of *D. officinale*. Based on a comparison of leaf and stem transcriptomes, our results reveal the presence of several GT, TF and sugar transporter genes. In addition, the genes encoding GT and Ces undergo a distinct degree of alternative splicing. Both transcriptional and post-transcriptional regulation may be involved in the synthesis of polysaccharides. We also report the differential expression of genes *DoSWEET4* and *DoSUT1* in the leaves and stems of *D. officinale*, and note that these sugar transporting genes should be targets for identifying quality polysaccharide production in medicinal plants. The results of this study reveal a new direction for investigations into polysaccharide accumulation in medicinal plants, via the process of sugar translocation in *D. officinale*.

## Supplementary Materials

The following are available online at www.mdpi.com/2073-4425/8/10/257/s1. Figure S1 Prediction of transmembrane helices in SWEETs and SUT from *D. officinale.* Table S1 Summary of the differentially expressed genes between leaves and stems. Table S2 Summary of putative SWEETs in *D. officinale*.

## Availability of Materials and Data

The datasets generated during the current study are available in the NCBI database (the accession number SRP094520 of bioproject PRJNA356162).
