# Supplementary material for: Hybrid Sequencing of Full-Length cDNA Transcripts of Stems and Leaves in Dendrobium officinale
Source: Genes (Basel). 2017 Oct 5;8(10):257. doi: 10.3390/genes8100257 (PMC5664107; doi:10.3390/genes8100257)
Supplement: Supplementary file 1 [file genes-08-00257-s001.zip › genes-223033- supplementary revised/genes-223033-Supplementary Information.docx]

**Supplementary Information**

**Table S1 Summary of the differentially expressed genes between leaves and stems.**

**Table S2 Summary of the putative *SWEETs* in *D. officinale*.**

| **Gene_id** | **Annotation** | **Length (aa)** | **Total** |
| --- | --- | --- | --- |
| TR85012 | SWEET1 | 256 | full |
| TR68775 | SWEET2a | 194 | partial |
| TR68226 | SWEET4 | 248 | full |
| TR67383 | SWEET14a | 265 | full |
| TR76199 | SWEET14b | 266 | full |
| TR77495 | SWEET15 | 241 | partial |
| TR61374 | SWEET16 | 190 | partial |
| TR73618 | SWEET17 | 253 | full |

**Figure S1** Prediction of transmembrane helices in SWEETs and SUT from *D. officinale*. The TMHMM2.0 program (www.cbs.dtu.dk/services/TMHMM) was used to predict transmembrane helices in DoSWEET1, DoSWEET4, DoSWEET14a, DoSWEET14b, DoSWEET17 and DoSUT1.

**
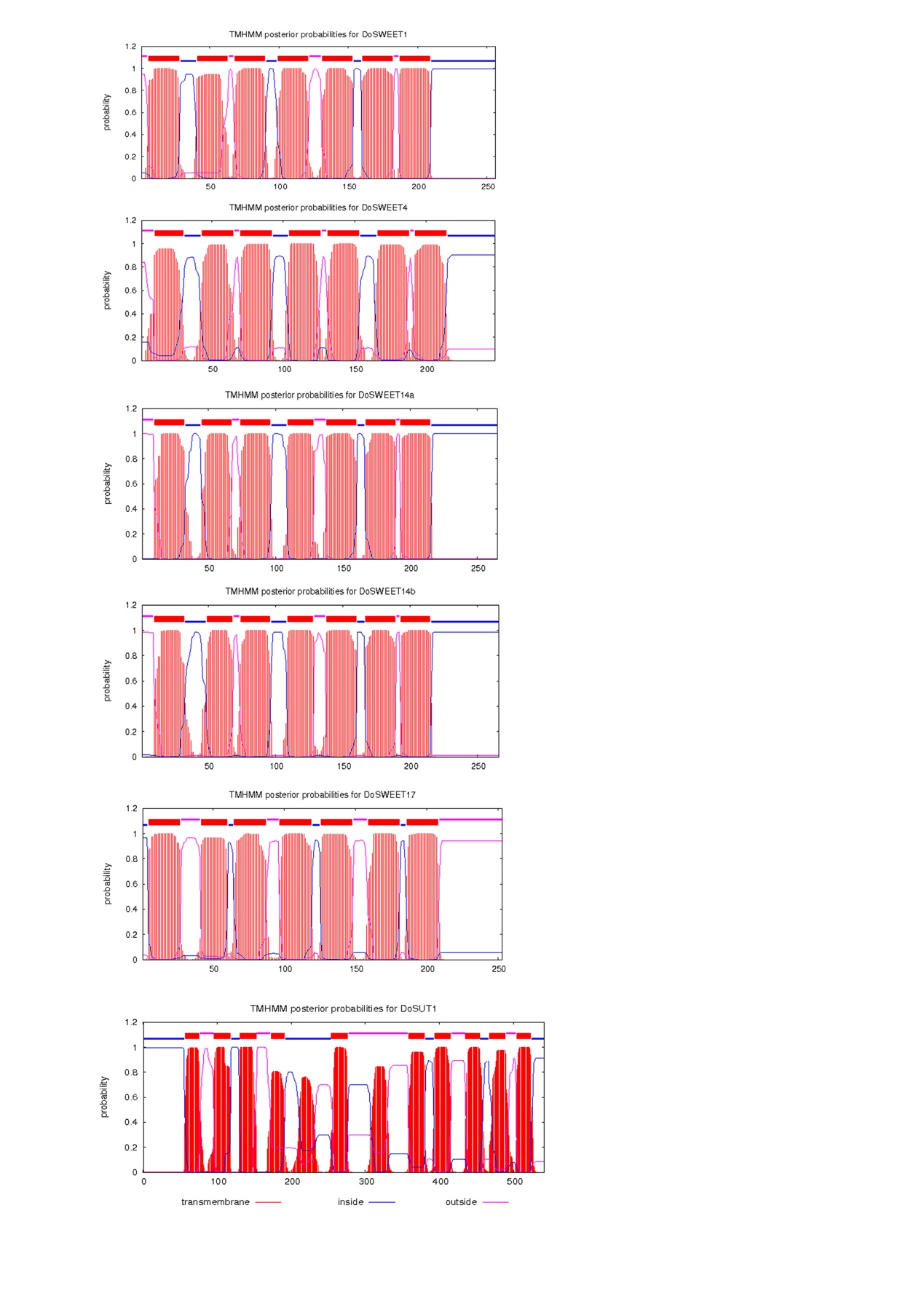
**
